# The effect of SSRIs on Semen quality: A systematic review and meta-analysis

**DOI:** 10.3389/fphar.2022.911489

**Published:** 2022-09-14

**Authors:** Jiarong Xu, Kancheng He, Yihong Zhou, Liangyu Zhao, Yuteng Lin, Zihao Huang, Nengqing Xie, Jihui Yue, Yuxin Tang

**Affiliations:** ^1^ Department of Urology, The Fifth Affiliated Hospital of Sun Yat-sen UniversityZhuhai, Zhuhai, China; ^2^ Guangdong Provincial Key Laboratory of Biomedical Imaging, The Fifth Affiliated Hospital of Sun Yat-sen University, Zhuhai, China; ^3^ Department of Psychiatry, The Fifth Affiliated Hospital of Sun Yat-sen University, Zhuhai, China

**Keywords:** SSRIs, semen quality, depression, premature ejaculation, meta-analysis

## Abstract

Selective serotonin reuptake inhibitors (SSRIs) are widely used for a variety of diseases, and their impact on semen quality is unclear. We performed a systematic search in PubMed and Embase, and after a strict screening, we included 4 studies with a total of 222 male participants. In result, SSRIs reduced normal sperm morphology (95% CI [−16.29, −3.77], *p* = 0.002), sperm concentration (95%CI [−43.88, −4.18], *p* = 0.02), sperm motility (95%CI [−23.46, −0.47], *p* = 0.04) and sperm DNA fragmentation index (DFI) (95% CI [6.66,21.93], *p* = 0.0002), without a statistically significant effect on semen volume (95%CI [−0.75,0.65], *p* = 0.89). Moreover, the impact on both sperm morphology and sperm concentration were observed within the 3-month period of SSRIs use. In general, our meta-analysis showed that SSRIs have a negative effect on semen quality. More larger, randomized, well-controlled clinical studies should be conducted to support our conclusion.

## Introduction

Selective serotonin reuptake inhibitors (SSRIs), including citalopram, escitalopram, fluvoxamine, paroxetine, fluoxetine, sertraline and dapoxetine, are a kind of drugs that have been used for many years (Atmaca, 2020). SSRIs can limit serotonin reabsorption into presynaptic cells, which increases serotonin levels in the synaptic gap and enhances extracellular serotonin activity (Warden and Fuchs, 2016; Bhattacharyya et al., 2019). They are widely used for the treatment of depression, obsessive-compulsive disorder, generalized anxiety, posttraumatic stress disorder, chronic pain, premenstrual dysphoric disorders and fibromyalgia (Koura et al., 2019). Because of their significant curative effect and high safety profile, SSRIs have been front-line pharmacotherapies in the treatment of depression for a long time (Cipriani et al., 2018). Additionally, SSRIs can also be used as a medicine for premature ejaculation because of the reversible sexual side effects (Giuliano and Clement, 2012; Bala et al., 2018).

Infertility is defined as failure to conceive after 12 months of regular and unprotected sexual intercourse (Zegers-Hochschild et al., 2017). It is estimated to affect 8–12% of couples of reproductive age worldwide, of which male causes account for approximately 50% of infertility case (Ombelet et al., 2008). Currently, the assessment of male infertility depends mainly on the analysis of semen quality indicators, such as sperm concentration, morphology, motility and the DNA fragmentation index (DFI) (Jodar et al., 2017; Agarwal et al., 2020; Agarwal et al., 2021). It has been reported that low DNA integrity in semen can reduce fecundability (Bungum et al., 2011). In addition, the problem of semen quality decline is also serious globally. In a meta-analysis including 185 articles and 42,000 people, semen quality was shown to have decreased in the past 40 years (Levine et al., 2017). Thus, we should pay more attention to observing the changes in semen quality and evaluating what truly affects sperm.

SSRIs can produce some side effects, including nausea, headaches, weight gain, erectile dysfunction and diminished libido (Csoka et al., 2008; Malhi and Mann, 2018). Regarding female fertility, it was reported that during the 2nd and 3rd trimesters, women using SSRIs may experience preterm birth and have low-birthweight infants (Huybrechts et al., 2014). Furthermore, Grigoriadis et al. (2013) concluded that SSRIs use while pregnant can increase the risk of congenital abnormalities of the heart. However, little attention has been given to the effects of SSRIs on semen quality in males. Studies explaining the relationship between SSRIs and semen quality are very limited, and the final conclusion remains unclear and controversial. For example, in a prospective study (Tanrikut et al., 2010), Tanrikut et al. assessed the effect of paroxetine on sperm parameters and the DFI measured by deoxyuridine-5′-triphosphate biotin nick end labeling (TUNEL) assays in 35 healthy male volunteers (mean age 34 years, range 19–58) who used therapeutic paroxetine for 5 weeks. They found that the patients treated with paroxetine had a significant increase in the DFI, while there were no significant changes in sperm parameters in their study. In contrast, Yland et al. (2021) reported that the recent use of psychotropic medications can decrease sperm concentration, sperm motility, sperm count and total motile sperm count.

Therefore, to distinctly study male fertility after treatment with SSRIs and provide better fertility guidance, we conducted a meta-analysis to evaluate whether SSRIs can influence semen quality.

## Methods

### Search strategy and study selection

**FIGURE 1:**
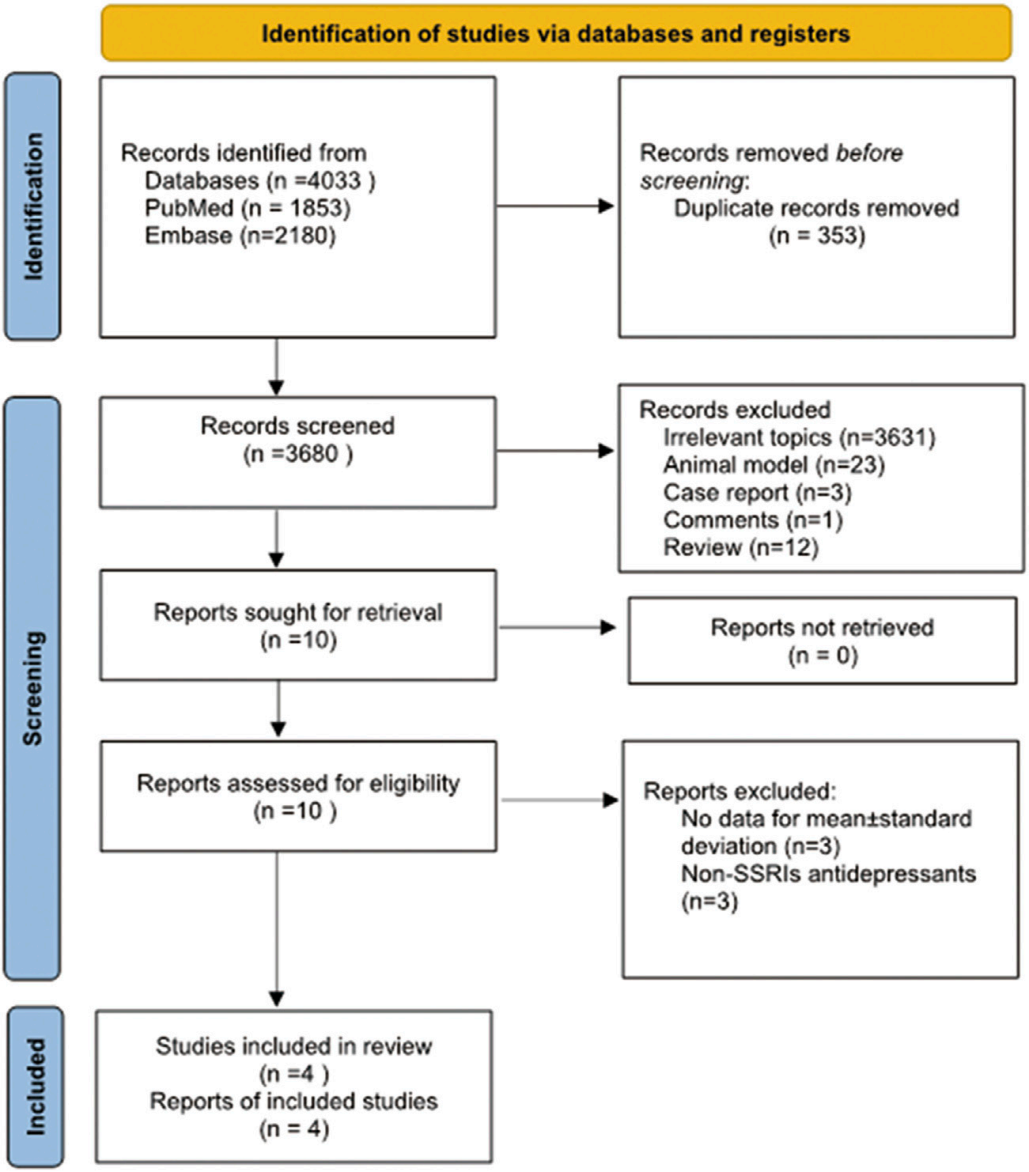
Articles search and screening process.

A literature search was conducted in PubMed and Embase from inception until 2 January 2022, using the following search terms: “SSRIs” or “selective serotonin reuptake inhibitors” or “antidepressants” or “psychotropic medications” or “depression” or “depressive symptom” or “emotional depression” or “premature ejaculation” and “semen” or “sperm” or “male infertility” or “sperm parameters” or “spermatozoon.” We also reviewed the related references within the included literature for studies that may have been overlooked in the search. After removing 353 duplicate publications, this search returned 3680 articles. The screening and selection process was conducted independently by three authors (Xu and He and Zhou). All the included studies met the following criteria: 1) studies with designs that were prospective or retrospective; 2) studies about the effect of SSRIs on semen parameters; 3) semen parameters were measured before and after SSRIs treatment, and 4) treatment was administered daily continuously. We excluded the following types of studies: 1) reviews; 2) case reports; 3) animal experiments; 4) comments; 5) studies about non-SSRIs antidepressants (e.g., monoamine oxidase inhibitors, tricyclic antidepressants); 6) semen parameters were not provided as mean ± standard deviation, and 7) participants with erectile dysfunction, asthenospermia, spermatogenesis disorders and other disorders affecting fertility. Articles written in English were chosen based on the language selection criteria. Three authors (Xu and He and Zhou) independently assessed the primary literature by assessing titles and abstracts and then identified the final relevant studies based on the included criteria. The article search and screening process are shown in Figure 1.

### Data extraction

Three authors (Xu, He, and Zhou) extracted data and information from the final studies, such as the country, publication year, patient age, study type, disease, sample size, type of SSRIs, dose of SSRIs, treatment duration, treatment period, semen volume, total sperm count, sperm concentration, sperm motility, normal sperm morphology ratio and the sperm DFI. We used the Newcastle–Ottawa Scale (NOS) (Stang, 2010) to assess the quality of the included studies.

### Statistical analysis

Review Manager 5.3 (RevMan 5.3) was employed to conduct all statistical analyses. For all analyses, a *p* value less than 0.05 was considered statistically significant. Data on semen volume, normal sperm morphology, sperm concentration, sperm motility and the sperm DFI were obtained from the final studies and using the mean ± standard deviation with their 95% confidence intervals (CIs). Standard Cochran’s Q test and I^2^ statistics were used to identify heterogeneity among the included studies. An I^2^ statistics value > 50% indicated significant heterogeneity. When heterogeneity was significant, we will present the results with the random effects model and explored the potential influential variables among the included studies and pooled the results in a subgroup analysis. Subgroup analyses were performed according to duration of treatment and different diseases. The mean and standard deviation were used as measurements with the random effects model in the subgroup analysis.

## Results

### Study characteristics

The essential information of all included articles is presented in Table 1 [(Safarinejad, 2008; Koyuncu et al., 2011; Akasheh et al., 2014; Korshunov et al., 2015)]. The 4 included studies were published from 2008 to 2015 with 222 participants. Among these studies, 2 were performed in Iran, 1 in Russia, and 1 in Turkey. In the 4 included articles, SSRIs were used for treatment daily of depression or premature ejaculation. All included studies had either a prospective or retrospective design and provided the mean ± standard deviation. The NOS was used to assess the methodological quality of the included studies, which ranged from 7 to 8 (Table 2).

**TABLE 1:** Basic characteristic of the included studies.

First author (year	Study region	Disease	Number of experimental group/control group	SSRIs type	Study design
Safarinejad 2008	Iran	Depression	74/44	Citalopram Escitalopram Fluoxetine Paroxetine Sertraline	Retrospective study
Koyuncu 2011	Turkey	Premature ejaculation	25/Self control	Escitalopram	Prospective syudy
Akasheh 2014	Iran	Premature ejaculation	30/30	Sertraline	Prospective study
Korshunov 2015	Russia	Depression	19/Self control	Fluoxetine	Prospective study

**TABLE 2:** Newcastle-Ottawa Scale for the included studies.

First author (year)	Selection	Comparability	Assessment of outcome	Total quality scores
Safarinejad 2008	**	**	***	7
Koyuncu 2011	***	**	***	8
Akasheh 2014	***	**	**	7
Korshunov 2015	***	**	***	8

### SSRIs and normal sperm morphology

**FIGURE 2:**
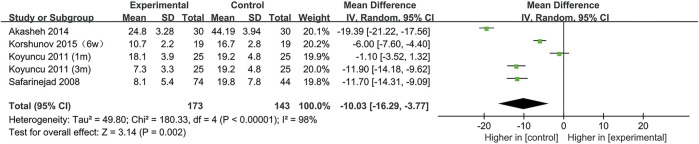
Forest plot for the association between SSRIs used and normal sperm morphology.

All included studies provided data on the percentage of sperm with normal morphology. During our analysis, we found that normal sperm morphology was significantly reduced after SSRIs use (95% CI [−16.29, −3.77], *p* = 0.002) (Figure 2).

**FIGURE 3:**
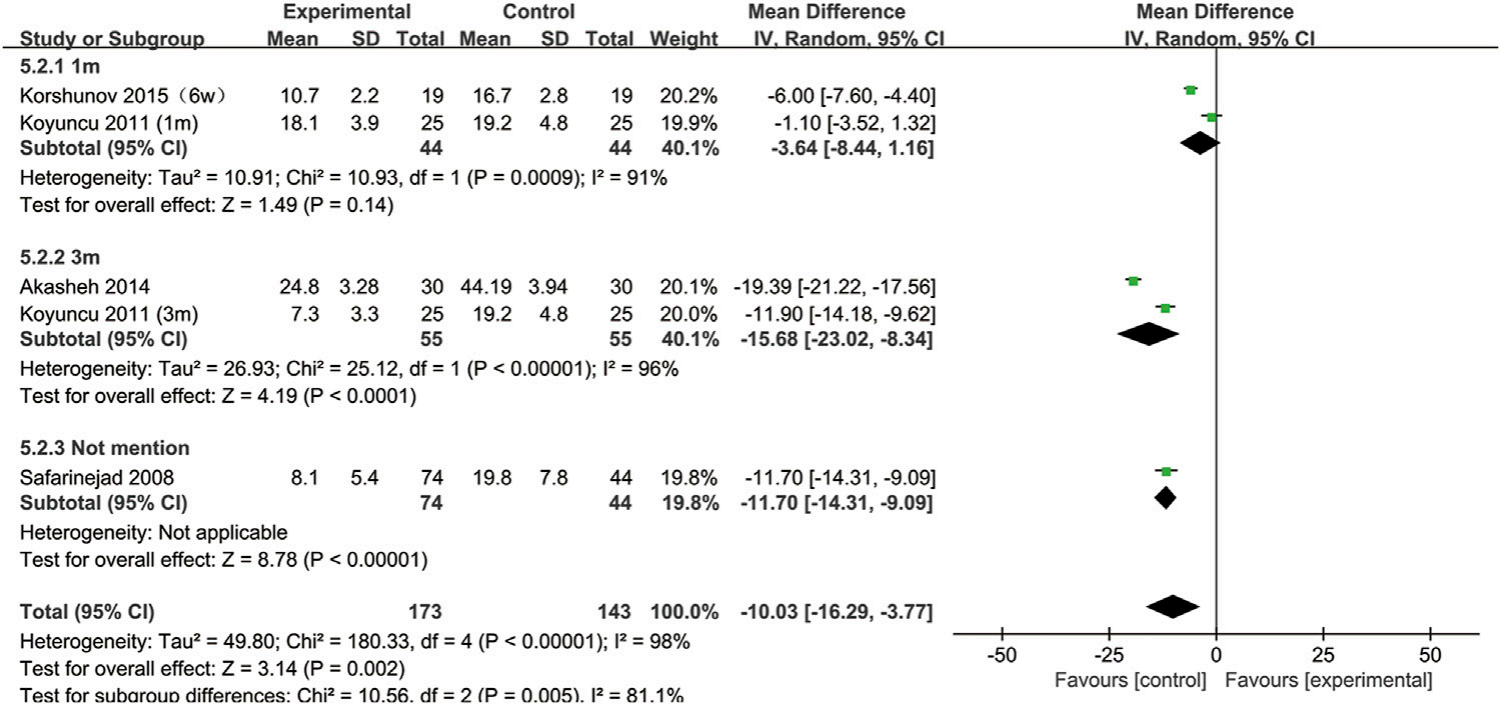
Subgroup of the association between normal sperm morphology and different SSRIs treatment duration.

**FIGURE 4:**
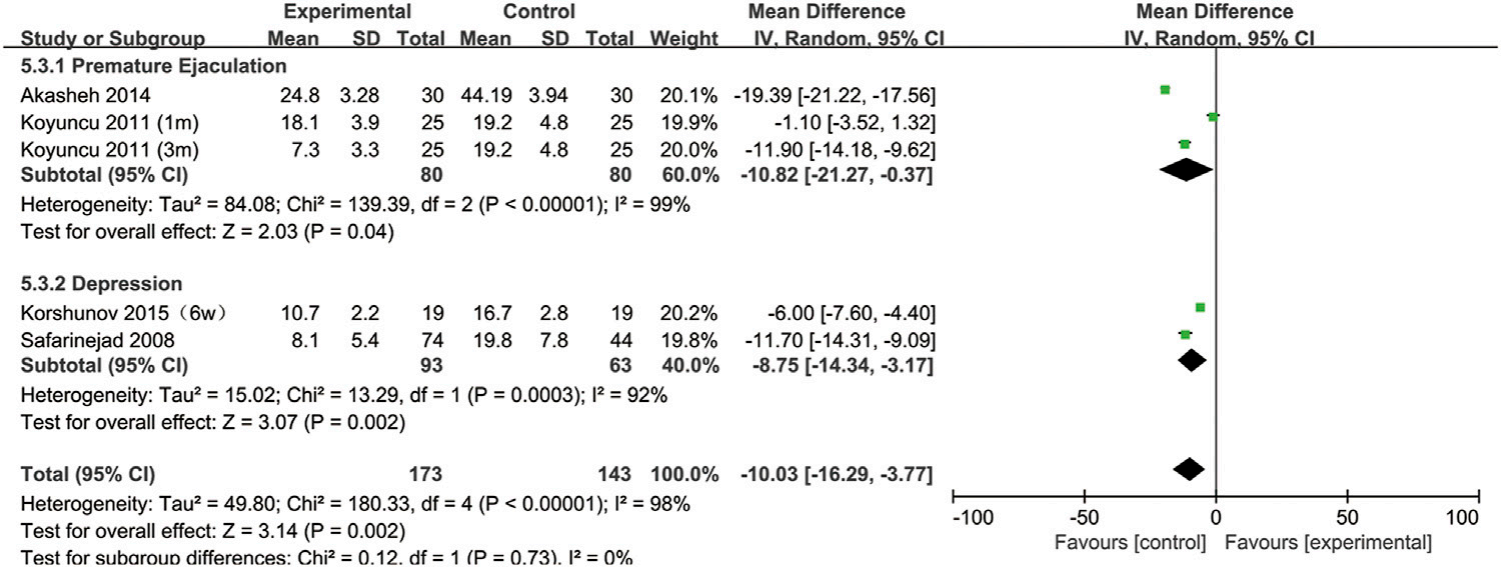
Subgroup of the association between normal sperm morphology and different basic disease.

Furthermore, to understand the relationship between the change in normal sperm morphology and the treatment duration and the type of disease, we performed a subgroup analysis. We included the articles with SSRIs treatment for 6 weeks in the same subgroup as those with treatment for 1 month. As shown in Figure 3, the percentage of normal sperm morphology was significantly reduced with an SSRIs treatment duration of 3 months (95% CI [−23.02, −8.34] (*p* < 0.0001)) compared with 1 month of treatment (95% CI [−8.44,1.16] (*p* = 0.14)). In Figure 4, we discovered that SSRIs treatment impacted sperm morphology in both patients with depression (95% CI [−14.34, −3.17] (*p* = 0.002)) and patients with premature ejaculation (95% CI [−21.27, −0.37] (*p* = 0.04)).

## SSRIs and sperm concentration

**FIGURE 5:**
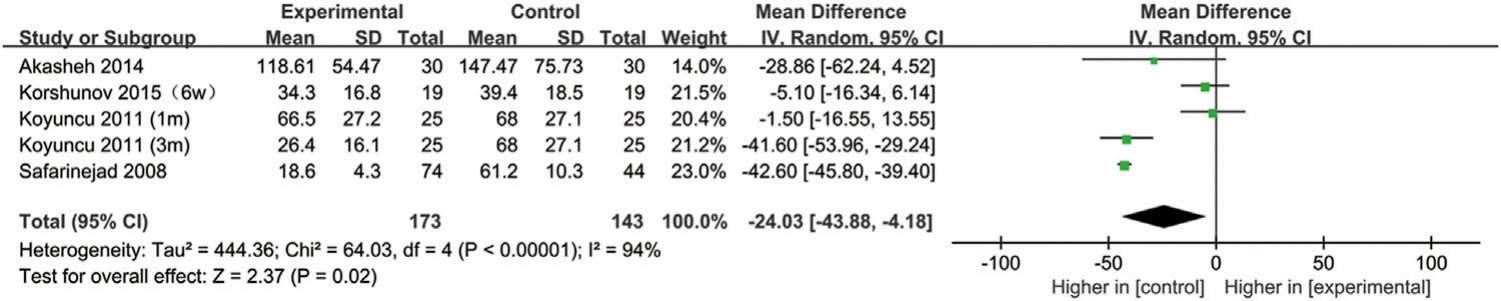
Forest plot for the association between SSRIs used and sperm concentration.

**FIGURE 6:**
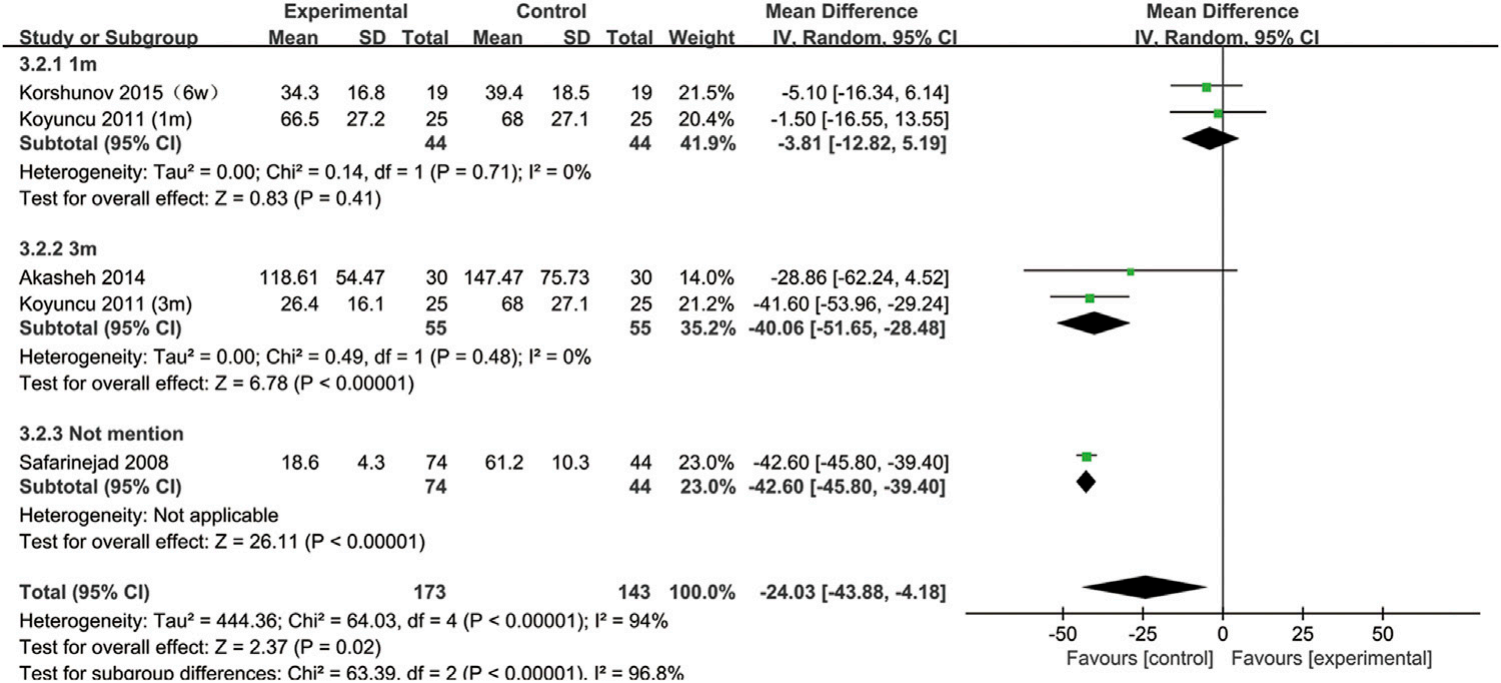
Subgroup of the association between sperm concentration and different SSRIs treatment duration.

**FIGURE 7:**
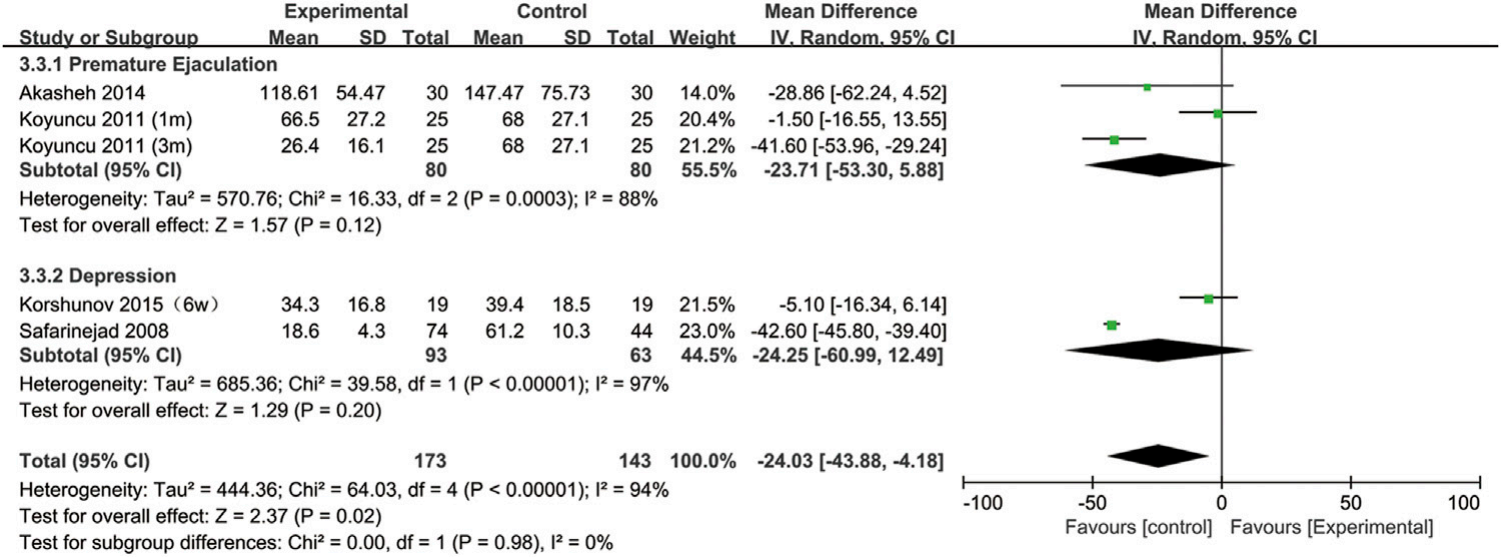
Subgroup of the association between sperm concentration and different basic disease.

All included studies provided data on the semen concentration. The sperm concentration in the experimental group was significantly lower than that in the control group (95% CI [−43.88, −4.18], *p* = 0.02) (Figure 5). In the subgroup analysis of SSRIs, we found that sperm concentration was reduced after SSRIs treatment for 3 months (95% CI [−51.65, −28.48], *p* < 0.00001), while 1 month of SSRIs treatment did not affect sperm concentration (95% CI [−12.82, 5.19], *p* = 0.41) (Figure 6). Moreover, in the subgroup analysis according to the disease type, neither depression (95% CI [−60.99, −12.49], *p* = 0.2) nor premature ejaculation (95% CI [−53.30,5.88], *p* = 0.12) drove the reduction in sperm concentration among patients who accepted SSRIs treatment (Figure 7).

## SSRIs and sperm motility

**FIGURE 8:**
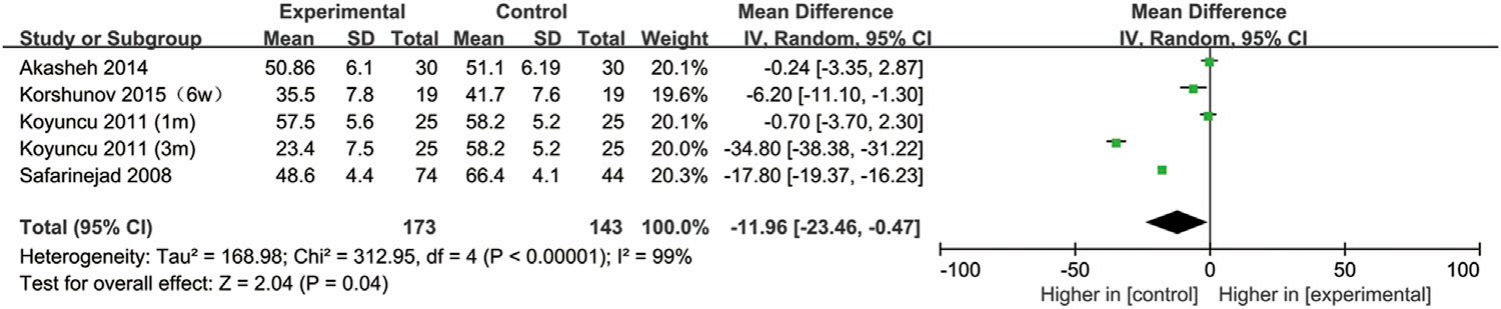
Forest plot for the association between SSRIs used and sperm motility.

**FIGURE 9:**
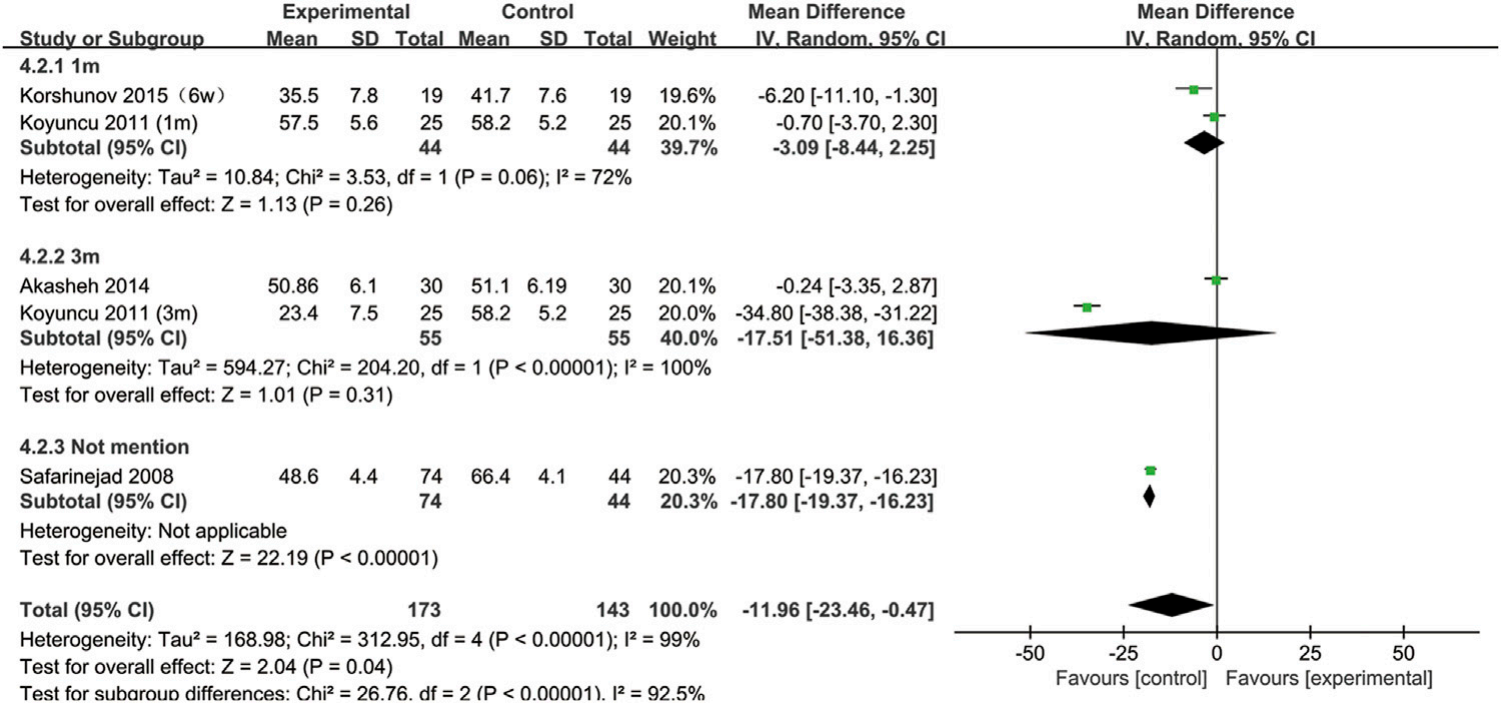
Subgroup of the association between sperm motility and different SSRIs treatment duration.

**FIGURE 10:**
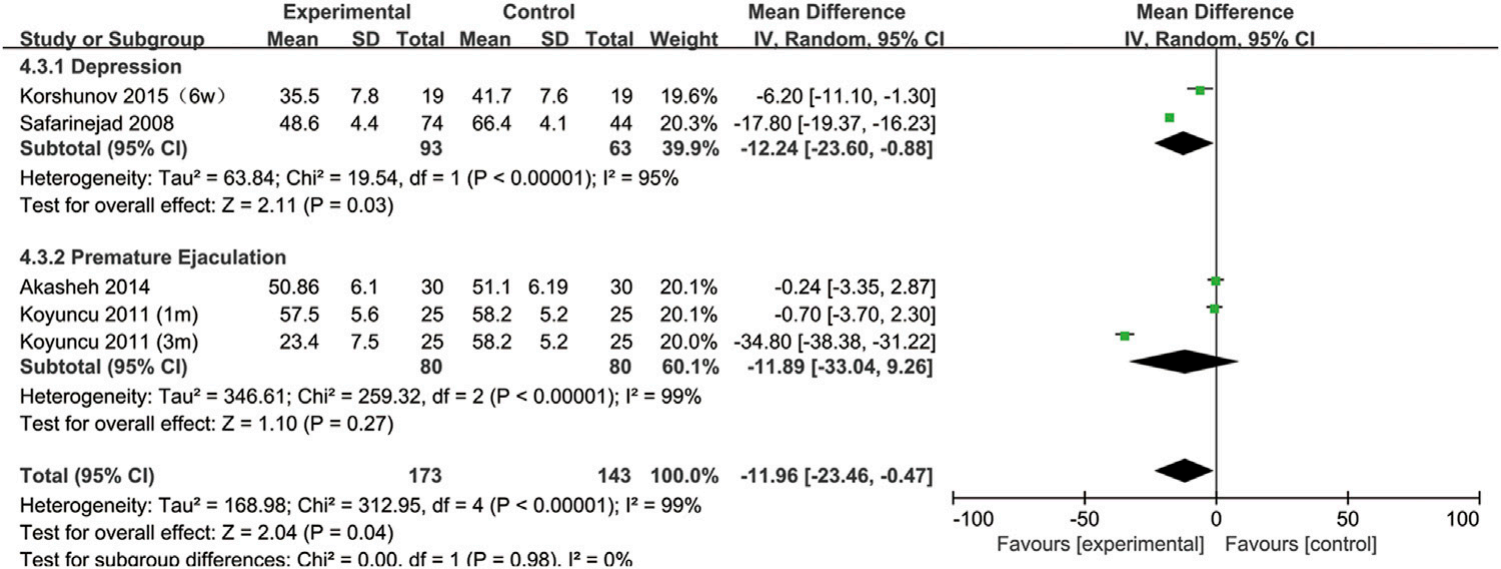
Subgroup of the association between sperm motility and different basic disease.

All included studies provided data on sperm motility. Figure 8 indicates that after SSRIs were used, sperm motility was notably reduced (95% CI [−23.46, −0.47], *p* = 0.04). However, when the subgroup analysis of treatment duration was conducted, we found that SSRIs had no effect on the group with a 1-month treatment duration (95% CI [−8.44,2.25], *p* = 0.26) or the group with a 3-months treatment duration (95% CI [-51.38,16.36], *p* = 0.31) (Figure 9). As shown in Figure 10, SSRIs had a negative effect on sperm motility in patients with depression (95% CI [−23.60, −0.88], *p* = 0.03) but not in patients with premature ejaculation (95% CI [−33.04, 9.26], *p* = 0.27).

### SSRIs and the DFI

**FIGURE 11:**
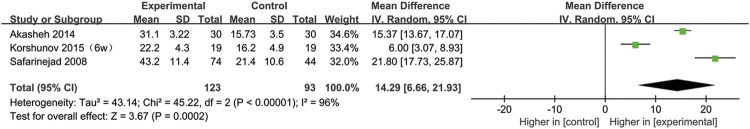
Forest plot for the association between SSRIs used and sperm DFI.

**FIGURE 12:**
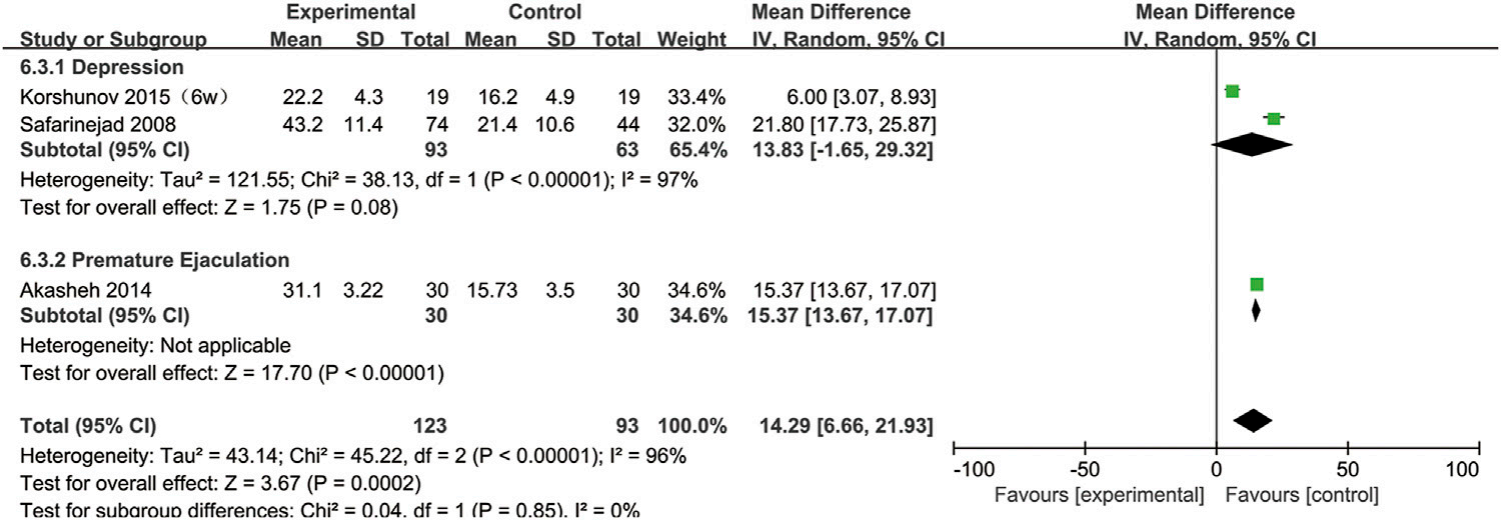
Subgroup of the association between sperm DFI and different basic disease.

Three studies provided sperm DFI data (Safarinejad, 2008; Akasheh et al., 2014; Korshunov et al., 2015). As shown in Figure 11, the DFI in the experimental group was significantly higher than that in the control group (95% CI [6.66, 21.93], *p* = 0.0002), suggesting that SSRIs had a negative effect on the DNA integrity. In the subgroup analysis of disease type, we found that SSRIs influenced the DFI in patients with premature ejaculation (95% CI [13.67,17.07], *p* < 0.00001) but n ot in patients with depression (95% CI [−1.65,29.32], *p* = 0.08) (Figure 12).

## SSRIs and semen volume

**FIGURE 13:**
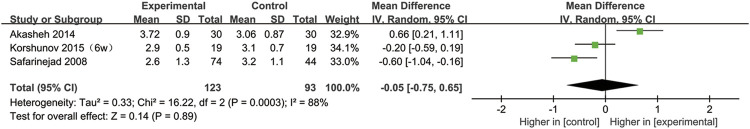
Forest plot for the association between SSRIs used and semen volume.

Data on semen volume were provided in 3 of the 4 included articles (Safarinejad, 2008; Akasheh et al., 2014; Korshunov et al., 2015). As shown in Figure 13, there was no significant influence on semen volume after SSRIs use (95% CI [-0.75, 0.65], *p* = 0.89).

## Assessment of heterogeneity

There was evidence of considerable heterogeneity in the DFI (I^2^ = 96%), normal sperm morphology (I^2^ = 98%), sperm concentration (I^2^ = 94%) and sperm motility (I^2^ = 99%). Subgroup analyses showed that the SSRIs treatment duration and basic diseases of the patients may have induced heterogeneity.

## Discussion

Semen quality can be affected by many factors, such as genetic factors, Y-chromosome microdeletions, lifestyle, environmental factors, smoking status, alcohol use, obesity, diseases such as varicocele and endocrine disruptors (Neto et al., 2016; Jensen et al., 2017; Virtanen et al., 2017)**.** With the annual increase in the proportion of male infertility, an increasing number of male patients are concerned about their semen quality. As we mentioned above, abnormal semen quality may cause infertility, so our meta-analysis suggesting the relationship between SSRIs and semen quality is quite meaningful and necessary.

The results indicate that SSRIs can affect sperm morphology, sperm concentration, sperm motility and sperm DNA integrity without a measurable influence on semen volume. Similarly, in a North American prospective cohort study (Yland et al., 2021), Yland et al. observed that recent use of psychotropic medications did not significantly change semen volume. Based on current knowledge, semen is made up of spermatozoa and seminal plasma and the semen volume is mainly determined by the amount of seminal plasma (Hamdi et al., 2020). A large amount of spermatozoa are produced in the seminiferous tubules in the testis, and seminal plasma is produced primarily by the epididymis, prostate, seminal vesicle and bulbourethral glands (Juyena and Stelletta, 2012; Lv et al., 2020). Thus, we consider that SSRIs probably only affect the production of spermatozoa in the testis and do not affect the production of epididymis fluid, prostatic fluid, seminal vesicle fluid or the bulbourethral glands fluid; in our analysis, semen volume did not significantly change after SSRIs use.

In the subgroup of treatment time, the effects on sperm morphology and density were all observed at 3 months but not at 1 month, which may be related to the period of spermatogenesis. As we know, the whole spermatogenic period requires approximately 72 days in humans (Muciaccia et al., 2013), so semen quality starts to change at least 72 days after SSRIs use. Therefore, 1 month of SSRIs use may not impact semen quality. However, Tanrikut et al. noted that paroxetine can induce abnormal sperm DNA fragmentation at 4 weeks, which concluded that the impact of SSRIs was on sperm transport rather than on spermatogenesis (Tanrikut et al., 2010). In our opinion, the 5-weeks exposure period may not completely reveal the effect of SSRIs on semen parameters and cannot be used to detect the impact on spermatogenesis. In the disease subgroups analyses, different disease subgroups had different results, probably owing to the limited number of included articles, so more literature needed in the future to explore the effect of SSRIS on semen quality in different disease subgroups.

To understand the relationship between SSRIs and semen quality, researchers began with experiments involving animals. Many studies have revealed that SSRIs have a negative effect on male fertility in animals. In an experimental rat model (Alzahrani, 2012), 15 rats were stochastically assigned to receive 3 oral doses of fluoxetine (2.6, 7.8, 13.0 mg/kg/day) for 5 days. The results showed that in the experimental group, the sperm count and sperm motility significantly decreased, and the proportion of abnormal sperm morphology significantly increased, which all existed in a dose-dependent manner. In a similar animal study (Ilgin et al., 2017)*,* 24 rats were divided into 3 groups to receive oral doses of citalopram hydrobromide (CTL) at 5, 10, and 20 mg/kg/day for 28 days. Sperm concentration, morphology and motility were measured by a computer-assisted sperm analysis system, and sperm DNA damage was observed by means of the comet assay. They found that in the CTL experimental groups, the sperm concentration and normal sperm morphology were significantly decreased, and sperm DNA damage was significantly increased. Bezerra et al. found that use of fluoxetine and sertraline in rats for a long period of time may reduce the sperm count and accelerate transit time through the epididymal cauda (Bezerra et al., 2019). Regarding the impact of SSRIs on offspring, Vieira et al. found that after pregnant rats received 7.5 mg/kg fluoxetine daily, both the weight of the seminal vesicle and the sperm count were decreased in male pups (Vieira et al., 2013).

In an vitro experimental study (Kumar et al., 2006), diluent SSRIs (paroxetine, fluoxetine, sertraline, citalopram and fluvoxamine) were mixed with human sperm samples. The authors observed that all the SSRIs antidepressants had spermicidal activity and that fluoxetine had the highest activity, which was probably related to the inhibition of oxidative phosphorylation in semen mitochondria to affect ATP synthesis. Moreover, this study also found that the addition of 5-hydroxytryptamine (5-HT) did not affect the spermicidal effect of SSRIs, suggesting that this effect was not mediated by 5-HT transporters.

Elnazer et al. reported a 30-year-old man with a diagnosis of mixed depressive and anxiety disorder who was treated with citalopram for almost 3 years (Elnazer and Baldwin, 2013). The patients had abnormal semen parameters during citalopram therapy, but after 4 months of discontinuation of citalopram, the sperm concentration, sperm motility, and sperm morphology all showed an obvious increase. Moreover, Tanrikut et al. reported early cases of oligozoospermia in two depressed patients (Tanrikut and Schlegel, 2007). One was treated with citalopram, and the other with sertraline. After discontinuing the SSRIs for several weeks, semen quality significantly improved, suggesting that the SSRIs impacted the transport of sperm rather than disrupting spermatogenesis.

At the same time, some articles that did not meet our inclusion criteria reported that SSRIs have a negative effect on semen quality. In a prospective study (Relwani et al., 2011), Relwani et al. summarized that sperm motility observably decreased when SSRIs were combined with other psychotropic drugs, but when SSRIs monotherapy was administered, sperm parameters did not significantly change. Moreover, Yland et al. (2021) observed that recent use of psychotropic medications had an impact on all semen quality parameters except semen volume.

However, these effects may be reversible. *T*anrikut et al. reported that sperm concentration and sperm motility were obviously increased after 1–2 months of the discontinuation of SSRIs (Tanrikut and Schlegel, 2007). Similarly, in another study, after stopping SSRIs treatment, semen quality reversed to baseline (Tanrikut et al., 2010). Therefore, during the treatment period, patients can temporarily delay attempts to conceive until the use of SSRIs is stopped.

The specific mechanisms by which SSRIs affect semen quality are still unclear. In our analysis, we concluded that the effect of SSRIs on sperm occurs over 3 months rather than 1 month, so we think SSRIs may disrupt the process of spermatogenesis, affecting semen quality. Elnazer et al. considered indoleamine 2,3-dioxygenase (IDO) to play a crucial role in the effect of antidepressant drugs on spermatogenesis, and the dysregulation of tryptophan metabolism may result in azoospermia (Elnazer and Baldwin, 2013). Ilgin et al. (2017) concluded that CTL induced abnormal semen quality by means of oxidative stress and hormonal changes. The specific and definite mechanisms of the effect of SSRIs on semen quality need to be confirmed by more experiments in the future.

There were several strengths of our meta-analysis. First, this is the first meta-analysis to reveal the relationship between SSRIs and semen quality. Second, we implemented a comprehensive search strategy in PubMed and Embase without publication type or publication date limitations and selected articles based on strict inclusion criteria. Third, we performed subgroup analyses of the treatment duration of SSRIs to comprehensively elucidate the effect of SSRIs on semen quality.

There were also some limitations of our meta-analysis. First, we only included 4 articles and a total of 222 participants in our meta-analysis, and the limited number of articles reduced the comprehensiveness of our analysis. Second, due to the incomplete data in the included studies, there was no subgroup analysis of the effect on the DFI, which is a vital indicator for guiding reproduction.

## Conclusion

Generally, our meta-analysis demonstrated that SSRIs have a statistically significant impairment on semen quality, such as sperm concentration, sperm morphology, sperm motility, and the DFI, but not on semen volume. Furthermore, the damage to sperm morphology and concentration were all observed in the 3-month period of SSRIs use but had no significant effect after 1 month of SSRIs use. Therefore, during the period of SSRIs treatment, patients of reproductive age may consider not conceiving or taking other drugs that have a lower potential for infertility. Finally, because the included articles were relatively limited, we need additional larger, randomized, well-controlled clinical studies to explain the relationship between SSRIs and semen quality.

## Data Availability

The original contributions presented in the study are included in the article/Supplementary Material, further inquiries can be directed to the corresponding authors.
